# Interbrain synchrony and its potential role in modulating the impact of traumatic events

**DOI:** 10.1038/s41398-025-03770-0

**Published:** 2025-11-28

**Authors:** Oded Mayo, Yael Molcho-Fisher, Yarden Avnor, Simone Shamay-Tsoory

**Affiliations:** School of Psychological Sciences, University of Haifa, Haifa, Israel

**Keywords:** Biomarkers, Neuroscience

## Abstract

Exposure to traumatic events has far-reaching effects on mental health. While social factors are known to exert buffering effects on trauma exposure, the underlying neural mechanisms that mediate these effects remain largely unexplored. Since interbrain synchrony is associated with social connectedness, we investigate whether individuals exhibiting a higher tendency for interbrain synchrony demonstrate reduced trauma-related symptoms. To examine whether interbrain synchrony assessed before trauma exposure moderated response to traumatic events, 98 participants who had previously participated in a hyperscanning functional near-infrared spectroscopy (fNIRS) study, which measured interbrain synchrony during an initial interaction with a stranger, were contacted following the terrorist attack in Israel on October 7th, 2023. They completed a questionnaire assessing their level of trauma exposure during the attack. As expected, the level of exposure to traumatic events was positively associated with PTSD, depression, and general psychiatric symptoms. As hypothesized, this association was moderated by interbrain synchrony, such that the greater the interbrain synchronization of brain activity in multiple brain regions, the weaker the association between exposure to traumatic events and symptoms reported by these individuals. This effect was most prominent in the left pre-motor cortex, which is part of the observation-execution system. These findings suggest that increased spontaneous interbrain synchronization during free conversations serves as a marker of social adaptation to the adversities of trauma and moderates the effects of traumatic exposure. Considering that interbrain synchrony facilitates social connection, these findings hold that individuals with a stronger predisposition for social connection are better equipped to cope with trauma.

## Introduction

Exposure to traumatic and highly stressful events exerts a profound impact on mental health and trauma-related psychopathology [1, 2]. Exposure to direct or indirect life-threatening events can result not only in heightened levels of post-traumatic stress disorder (PTSD) but also in a broader spectrum of adverse mental health outcomes, including elevated risks of various psychiatric disorders [1–5]. A dramatic increase in the prevalence of PTSD, depression, and anxiety disorders is particularly reported following life-threatening situations such as terrorist attacks [6–9].

However, individual responses to traumatic events vary considerably, depending on a range of personal and social factors, including biological risk factors such as dysfunctions in key brain regions like the amygdala, hippocampus, and ventromedial prefrontal cortex (vmPFC) [1, 2, 10–12]. Far less is known about the neural mechanisms underlying social behaviors that modulate the impact of exposure to traumatic events. Social relationships play a critical role in buffering against the negative effects of trauma by providing emotional, psychological, and social support [13–15]. For example, perceived social support from family, friends, and significant others mitigates acute anxiety symptoms among civilians who were exposed to missile attacks [16].

Considering that interbrain synchrony, the temporal alignment of neural activities between interacting partners [17–19], was shown to play a key role in supporting interpersonal connectedness [20], it may serve as a potentially promising biomarker of interpersonal interaction quality. Interbrain synchrony reflects the coupling of cognitive processes (e.g., attentional states), mutual predictions, and understanding [21–24]. The role of interbrain synchrony can also be understood within the broader framework of biobehavioral synchrony [25]. Through these synchronous interactions, especially during early caregiving, individuals develop emotion regulation capacities and stress coping mechanisms [25, 26]. Embedding interbrain synchrony within this theoretical context highlights its potential as a resilience factor, not just a measure of momentary coordination. Importantly, interbrain synchrony captures emergent neural processes that arise between individuals, not within them. While traditional studies of resilience have focused on individual brain responses, such as reactivity in the social brain or emotion regulation networks [27] these approaches overlook the dynamic, reciprocal nature of social interaction. A dyadic framework allows us to examine how people co-regulate one another in real time, especially in the face of adversity.

Particularly, interbrain synchrony within the observation-execution network encompassing the inferior parietal lobule, inferior frontal gyrus (IFG), and premotor cortices, has been implicated in facilitating social alignment [28]. Specifically, this system is related to the alignment of emotional processes, movements, and cognitive processes that are related to feelings of social closeness. The IFG plays a role in behavioral synchrony in both single-brain [29, 30] and two-brains studies [31], with interbrain synchrony observed during cooperative tasks [32]. In addition to the IFG, the premotor cortices were shown to be part of the mirror neuron systems, in which actions and observation of actions show similar patterns of activity [33]. Increased activity in the premotor cortex was associated with mutual action and cooperation [34]. Another relevant brain region is the dorsolateral prefrontal cortex (dlPFC), which has a central role in executive functions [35] and emotional regulation [36–38]. Overall, interbrain synchrony in these areas has been found related to social communication [39] and is considered a biomarker of closeness and cooperation [40].

Notably, some individuals synchronize more than others regardless of their partner or situation and are seen as more socially attractive due to better adaptation to social interactions [41, 42]. This suggests that interpersonal synchrony reflects both an emergent property of interaction and a personal tendency [24, 41–43]. A recent study found that pre-pandemic synchrony in heart rate moderated the impact of perceived closeness during lockdown [43], providing evidence that physiological synchrony may facilitate adaptation to stress and trauma.

Therefore, investigating interbrain synchrony provides an opportunity to move beyond static, individual-level traits like perceived support and examine how neural coordination with others may actively shape resilience. This dyadic lens helps reveal real-time interpersonal mechanisms that individual-level measures cannot capture.

The current study aimed to explore interbrain synchrony as a moderating factor in the impact of exposure to traumatic events. We recruited participants exposed to the events of October 7th, 2023 in Israel, who had previously participated in an fNIRS study measuring interbrain synchrony during a first interaction with a stranger, a reliable measure of social connection [44]. This setup allowed us to assess rapport-building in real time and examine individual differences in the tendency for interbrain synchrony and its link to stress resilience. Our first hypothesis was that exposure to trauma would correlate with increased rates of PTSD, depression, and general psychiatric symptoms, replicating previous findings. Second, we hypothesized that individuals who show a greater tendency to synchronize their brain activity in the IFG, premotor cortices, and dlPFC, will demonstrate increased resilience in response to traumatic and stressful events. Namely, for these individuals, the association between the level of exposure and trauma-related psychopathology will be weaker. Finally, we examined which brain regions show a moderating effect on trauma symptom development with particular emphasis on the IFG and the premotor cortex, regions known to be part of the observation-execution system.

## Materials and methods

### Participants

The sample included 174 potential participants who participated in an earlier interaction study and were contacted via email to fill out online questionnaires. Of those, 111 participants completed the questionnaires. Crucially, there were no differences between responders and non-responders in age (t = 0.53, p = 0.60), symptoms of depression (t = 0.41, p = 0.68), and post-traumatic symptoms (t = 1.09, p = 0.28). Due to technical difficulties in the earlier study, neuroimaging data were not available for thirteen individuals, thus resulting in a final sample of 98 participants. Our current sample size in the study was thus predetermined by the original study; hence, we could not decide on the sample size based on an a priori power analysis, and a post hoc power analysis could not provide additional meaningful information [45]. Yet the current sample size is similar to other fNIRS studies that have been published recently [46–49].

73.5% (N = 72) of our sample were women, and 26.5% were men (N = 26). Their mean age was 25.8 years (SD = 3.59, range = 19–38). The native tongue of 90.82% was Hebrew (N = 89), while Arabic was the native tongue of the rest (9.18%, N = 9). The questionnaires were filled out between 29/12/2023 and 04/02/2024. The mean time between the original study and the current study was 530 days (SD = 327 range = 107–1135 days).

### Self-report measures

#### Measurement of post-traumatic symptomatology

Overall PTSD symptoms were assessed using the PTSD Checklist (PCL-5) [50]. This 20-item questionnaire, known for its high psychometric properties, validity, and test-retest reliability [51], requires participants to indicate the frequency with which they experienced specific PTSD symptoms in the past month, on a scale of 1–6 (1 = never experienced, 6 = experienced a significant amount).

#### General psychiatric symptoms

General psychiatric symptoms were assessed using the Brief Symptom Inventory (BSI) [52]. This scale consists of [50] items covering nine symptom dimensions (Somatization, Obsession-Compulsion, Interpersonal Sensitivity, Depression, Anxiety, Hostility, Phobic Anxiety, Paranoid Ideation, and Psychoticism) and three global indices of distress (Global Severity Index, Positive Symptom Distress Index, and Positive Symptom Total). Additionally, the BSI includes subscales that assess depression and anxiety, helping to identify particular areas of concern and providing an indication of overall psychological distress.

#### Symptoms of depression

The symptoms of depression were measured using the Beck Depression Inventory-II (BDI-II) [50]. This 21-question multiple-choice self-report inventory assesses the severity of depression with high discriminant validity that ensures that it does not correlate highly with measures of other psychiatric symptoms [53].

#### Exposure to stressful events following the October 7, 2023 massacre

This scale was developed to assess levels of exposure to traumatic events during and after the terror attack on October 7 in Israel and was based on a similar questionnaire [54]. The questionnaire includes questions referring to threats to life or physical safety, the need to hide from threats, harm to personal property, or the unavailability of basic services. For example: “I was in a place where my life was under serious threat”, “I had to find shelter in my home/safe room/bomb shelter due to a direct and immediate threat”, and “I was in a situation where my mental well-being was harmed or threatened”. The scale ranges from 1 (no exposure at all in the period since October 7)–6 (exposure almost every day, one time or more). Cronbach alpha was 0.81, and McDonald’s omega was 0.83. The complete questionnaire can be found in the supplementary file.

#### Perceived interpersonal closeness

We assessed participants’ perceived closeness to others using the Perceived Interpersonal Closeness Scale (PICS) [55]. Participants viewed a diagram of seven concentric circles representing increasing levels of closeness, from “self” (innermost) to “distant” (outermost), and placed each of 17 types of significant others (e.g., partner, parent, friend, colleague, pet) into the circle that best reflected their current relationship. An additional “not relevant” option was included. Following the scale’s guidelines, each placement was scored from 0 (“not relevant” or “distant”) to 5 (“self”), and scores were summed to create an overall closeness index ranging from 0–85. In this study, scores ranged from 0–76, with higher scores indicating greater perceived closeness across relationship types.

### Interbrain synchrony

#### Neural data acquisition

**Fig. 1:**
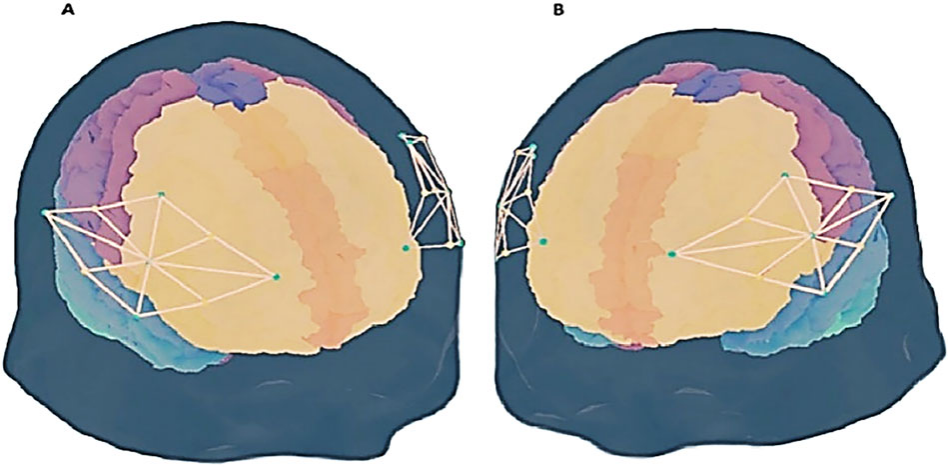
Brite24 fNIRS Optode Montage. The Brite24 fNIRS caps optode montage is illustrated relative to the EEG 10/20 system. Green dots represent transmitters and yellow dots represent receivers. A channel (orange line) is formed with one transmitter and one receiver, with overall 12 channels on each hemisphere (2 × 12). Channels cover the following regions of interest: bilateral dorsolateral prefrontal cortex, bilateral inferior frontal gyros, and bilateral pre-motor cortices. **A** Represents a right-side view of all available channels. **B** Represents a left-side view of all available channels.

The Brite-24 Artinis fNIRS system (Artinis Medical Systems, Els, Netherlands) was employed to gather continuous-wave fNIRS data from participants during the first meeting. The system utilized 4LED light sources emitting at 760 nm and 850 nm, which were detected by 5 light detectors, resulting in 12 measurement channels per hemisphere. Optical signals were recorded at a frequency of 25 Hz. The fNIRS head caps were positioned according to the international 10–20 coordinate system, covering bilateral IFGs, dlPFCs, and premotor cortices. Alignment with the 10–20 system was confirmed using a Polhemus Patriot 3D Digitizer (Polhemus, Colchester, Vermont, USA). For an illustration of the caps and optode montage see Fig. 1.

#### Neural data preprocessing

Preprocessing analysis was carried out using MATLAB (MathWorks, Natick, MA, USA) with the Homer3 software [56]. All fNIRS channels were manually inspected for the presence of a heartbeat signal (~1 Hz). Channels without detectable heartbeat were excluded from further analysis. All exclusion criteria (missing neuroimaging data; channels lacking a detectable heartbeat) were pre-specified before statistical analysis. The raw intensity values were converted to Optical Density (OD) using the modified Beer-Lambert law (MBLL). Motion artifacts were removed using standard deviation (SD) and amplitude change (AMP) thresholding over 0.5 s windows, along with a spline correction method to eliminate motion-induced spikes in the signal. A bandpass filter (0.01–0.5 Hz) was then applied to remove artifacts related to cardiovascular and pulmonary activity. Finally, the OD values were converted to Oxyhemoglobin (O2Hb) and deoxyhemoglobin (HHb) concentration values for subsequent analysis.

#### Clustering channels into regions of interest

Regions of Interest (ROIs) comprised six areas including the bilateral dlPFC, IFG, and Premotor cortices. Concentration values of pre-processed O2Hb from fNIRS channels covering each ROI were averaged per participant, creating a single data vector per ROI per participant.

#### Analysis of interbrain synchrony

Interbrain synchrony was computed with the Wavelet Transform Coherence (WTC) analysis using the WTC toolbox for Matlab [57]. Using a Morlet wavelet, the mother wavelet, WTC values of oxyhemoglobin (O2Hb) concentration were obtained. We used the O2Hb signal since it was found a more accurate and sensitive measure of changes in blood flow in fNIRS [58]. This Morlet function was constructed for wavelengths in the range of 33–66 sec. (~0.083–~0.1666 Hz), following the expected time range of the Blood Oxygenation Level Dependent (BOLD) signals, as part of the Hemodynamic Response Function [59]. Overall, we analyzed 6 ROI combinations including only homologous regions. To validate that interbrain synchrony reflected true interpersonal dynamics rather than spurious correlations, we additionally created pseudo-dyads by randomly pairing participants who had not interacted with each other. Interbrain synchrony values in real dyads were then compared against those in pseudo-dyads, serving as a control condition to test whether synchrony exceeded chance levels .

### Procedure

#### Ethics statement

The study was approved by the University of Haifa, Department of Psychology Ethics Committee (IRB approval number 086/23). All experimental protocols were performed in accordance with relevant guidelines and regulations, and all participants provided written informed consent prior to participation.

#### The first meeting paradigm

To assess the emergence of interbrain synchrony during the first interaction with a stranger, participants were recruited and then divided into pairs, matched based on sex, native tongue, and hand dominance. Upon arrival, participants, who had no prior acquaintance, were fitted with the fNIRS cups and were asked to have a five-minute conversation aimed at getting to know each other. The experimenter advised them to approach the conversation as they would when meeting someone new, suggesting they ask questions to learn more about the person in front of them, show interest in their personal life, and try to discover shared interests or hobbies. The researcher then informed the participants that she would leave the room during their conversation and that the discussion would conclude upon her return. The participants were instructed to talk in their native language, for their convenience.

**Fig. 2:**
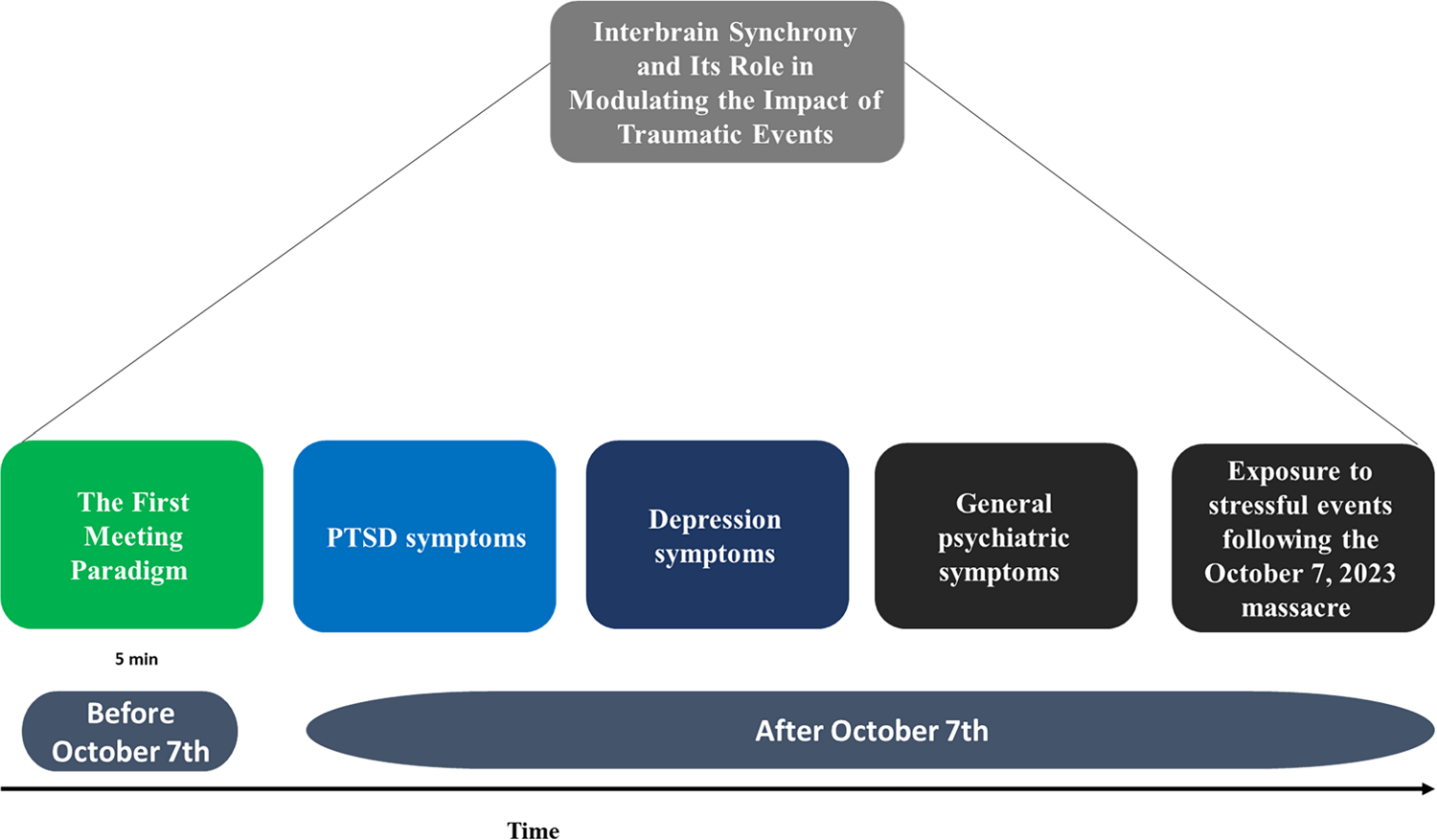
Study Design Overview. Illustration of the study design, including the first meeting paradigm that was conducted before October 7^th^ and questionnaires that were conducted after October 7^th^.

Participants of the original study received an invitation to the current online survey via email. The online survey, powered by Qualtrics, was conducted between 29/12/2023 and 04/02/2024. Each participant received a compensation of 20 NIS (approximately $5). Figure 2 illustrates the procedure.

### Statistical analysis

We used three mixed linear models, to examine the effect of exposure to trauma and interbrain synchrony on trauma-related psychopathology. Our analyses were done using R statistical software (Version 4.4.1) and Jamovi (2.4.11). The first model examined post-traumatic stress symptoms, the second examined depressive symptoms, and the third examined general psychiatric symptoms. For each of these models, we investigated the main effects of exposure to stressful events, interbrain synchrony, the region of interbrain synchrony (ROI), and the interactions between them. We also introduced the time since baseline measurement (in days) as a covariate in the model. We had three focal hypotheses for each model: (1) a main effect of exposure to stressful events, (2) an interaction between exposure and interbrain synchrony, and (3) a three-way interaction between exposure, interbrain synchrony, and ROI. Although the study was not preregistered, these hypotheses were specified a priori. To control for multiple comparisons, we applied false discovery rate (FDR) [60] correction separately within each ROI, but not across ROIs. This approach reflects our focus on testing interaction effects within predefined regions, without assuming a global correction across all regions.

To analyze the interaction effect of exposure to stressful events X interbrain synchrony, we plotted the interaction at the mean of the moderator (interbrain synchrony), 1 standard deviation above and below the mean of the moderator. Additionally, we conducted a simple slope analysis of the effect of exposure to stressful events at the mean of the moderator, 1 standard deviation above and below the mean of the moderator.

To analyze the three-way interaction exposure to stressful events X interbrain synchrony X ROI, we ran the model in each ROI separately.

Finally, to find whether the moderating effect of interbrain synchrony has a unique contribution, beyond general social support, we conducted two additional analyses: (1) we included PICS as a covariate in all of the models, and (2) we examined the association between interbrain synchrony and PICS (as a dependent variable). We also tested the effect of gender in our main models.

## Results

To provide an overview of the outcome variables in this study, we calculated the means standard deviations, and ranges for post-traumatic stress symptoms, depressive symptoms, and general psychiatric symptoms. The mean score for post-traumatic stress symptoms was M = 19.70, with a standard deviation of SD = 17.90 (range = 0–72). Depressive symptoms had a mean score of M = 14.3, with a standard deviation of SD = 12.10 (range = 0–50). Finally, psychiatric symptoms exhibited a mean of M = 61.60 and a standard deviation of SD = 44.10 (range = 0–194). We also calculated the interbrain synchrony scores across all ROIs: right IFG (M = 0.31, SD = 0.06), left IFG (M = 0.29, SD = 0.07), right dlPFC (M = 0.30, SD = 0.08), left dlPFC (M = 0.32, SD = 0.07), right premotor cortex(M = 0.31, SD = 0.12), left premotor cortex (M = 0.30, SD = 0.10). Real dyads exhibited significantly greater interbrain synchrony compared to pseudo-dyads across ROIs (F = 4.31, p = 0.04), supporting the validity of our synchrony measure.

To examine the relative effects of exposure to stressful events, interbrain synchrony, and the region of interbrain synchrony on post-traumatic stress symptoms, we created a mixed linear model that included these effects as well as the interactions between them. We also introduced the time since baseline measurement (in days) as a covariate in the model. Interbrain synchrony, exposure to stressful events, and the time since baseline measurement were all centered on their mean. Additionally, we assigned the random effect of the intercept of the couple. Our model equation is included in the supplementary file (Equation 1).

Results indicated that our model accounted for an overall 22.9% of the variance of post-traumatic stress symptoms. There was a main effect for exposure to stressful events (p < 0.001), and one interaction effect: exposure to stressful events X interbrain synchrony (p = 0.03). The interaction interbrain synchrony X Exposure to stressful events X ROI was not significant (for full details of the model see Table S1 in the supplementary file).

**Fig. 3:**
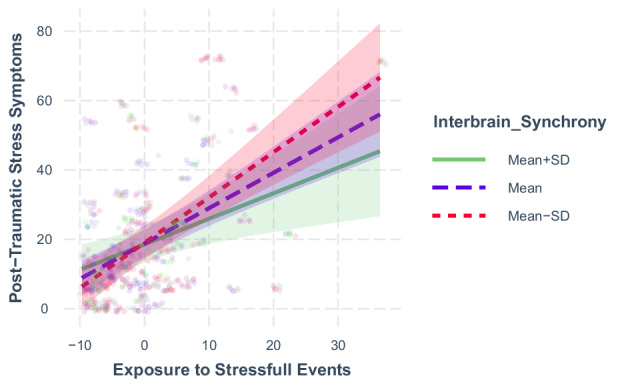
Stressful Event Exposure and PTSD Symptoms Across Levels of Interbrain Synchrony. Exposure to stressful events and post-traumatic stress symptoms in participants with Mean (t = 11.37, p < 0.001), High (t = 7.22, p < 0.001), and Low (t = .10.79, p < 0.001) interbrain synchrony. Dots are individual observations, and the shaded lines represent the confidence interval of the slopes.

Next, we investigated the interaction between interbrain synchrony and exposure to stressful events by plotting the interaction at the mean (+/- 1 standard deviation) of the moderator. A simple-slope analysis revealed that the effect of exposure to stressful events on post-traumatic stress symptoms was the highest when interbrain synchrony was low (estimate = 1.21, p < 0.001), and lower when interbrain synchrony was at the mean (estimate= 1.04, p < 0.001) and when interbrain synchrony was high (estimate = 0.87, p < 0.001). See Fig. 3.

Overall, these results indicate that exposure to traumatic events is positively associated with PTSD symptoms, such that the higher the exposure, the higher the level of the symptoms. Moreover, this association is moderated by pre-exposure interbrain synchrony, showing that individuals who generally show higher interbrain synchrony have a weaker association between exposure and symptoms.

Next, we explored the relative effects of exposure to stressful events, interbrain synchrony, and the region of interbrain synchrony on psychiatric symptoms. Our model equation is included in the supplementary file (Equation 2).

Our model accounted for an overall 24.3% of the variance of general psychiatric symptoms. There was a main effect for exposure to stressful events (p < 0.001), and two interaction effects: exposure to stressful events X interbrain synchrony (p = 0.04), and exposure to stressful events X interbrain synchrony X ROI (p = 0.04) (for full details of the model see Table S2 in the supplementary file).

**Fig. 4:**
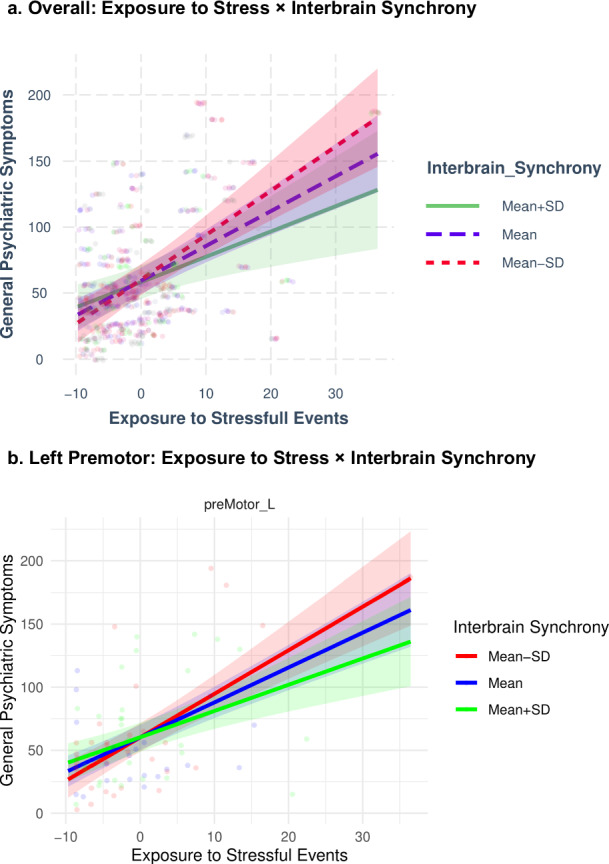
Stressful Event Exposure and General Psychiatric Symptoms Across Levels of Interbrain Synchrony. **a**. Exposure to stressful events and general psychiatric symptoms in participants with Mean (t = 12.19, p = < 0.001), High (t = 8.00, p = < 0.001), and Low (t = 11.32, p = < 0.001) interbrain synchrony. **b**. Exposure to stressful events and general psychiatric symptoms in participants with Mean, High, and Low interbrain synchrony in the left pre-motor cortex. Dots are individual observations, and the shaded lines represent the confidence interval of the slopes.

To further investigate the interaction interbrain synchrony X exposure to stressful events we plotted the interaction at the mean of the moderator (+/- 1 standard deviation). A simple-slope analysis showed that the effect of exposure to stressful events was the highest when interbrain synchrony was low (estimate = 0.86, p < 0.001), and lower when interbrain synchrony was at the mean (estimate = 0.75, p < 0.001) and when interbrain synchrony was high (estimate = 0.64, p < 0.001). See Fig. 4a.

Finally, we examine the three-way interaction exposure to stressful events X interbrain synchrony X ROI, by looking at the model in each ROI separately. This analysis revealed that while the main effect of exposure to stressful events was significant in each ROI (for full details of the model see Table S3 in the supplementary file), the interaction effect of exposure to stressful events X interbrain synchrony was significant only for the left premotor area (F = 5.07, p = 0.02). There was a trend towards significant interaction of the exposure to stressful events X interbrain synchrony, in the right (p = 0.09) and left (p = 0.07) IFG. In the left premotor area, it is evident that the slope of exposure to stressful events is steeper when interbrain synchrony is lower (Fig. 4b).

In summary, these results indicate that exposure to traumatic events is positively associated with general psychiatric symptoms, such that the higher the exposure, the higher the level of the symptoms. This association is moderated by interbrain synchrony pre-exposure to trauma, such that individuals who synchronized more show a weaker association between exposure and symptoms. Additionally, this interaction effect is stronger for synchrony in the left premotor area.

To examine the extent to which exposure affected symptoms of depression and whether its effect was moderated by interbrain synchrony, we explored the relative effects of exposure to stressful events, interbrain synchrony, and the region of interbrain synchrony on depression symptoms. Our model equation appears in the supplementary file (Equation 3).

Our model accounted for an overall 26.5% of the variance of depression symptoms. There was a main effect for exposure to stressful events (p < 0.001), and two interaction effects: exposure to stressful events X interbrain synchrony (p = 0.02), and exposure to stressful events X interbrain synchrony X ROI (p = 0.03) (for full details of the model see Table S4 in the supplementary file).

**Fig. 5:**
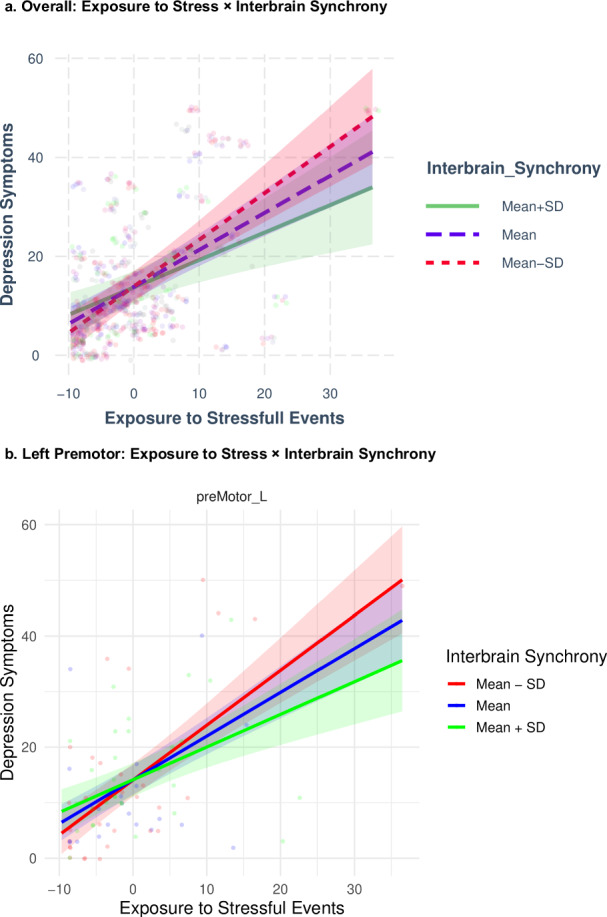
Stressful Event Exposure and Depression Symptoms Across Levels of Interbrain Synchrony. **a**. Exposure to stressful events and depression symptoms in participants with Mean (t = 13.29, p = < .001), High (t = 8.58, p = < 0.001), and Low (t = 12.53, p = < 0.001) interbrain synchrony. **b**. Exposure to stressful events and depression symptoms in participants with Mean, High, and Low interbrain synchrony in the left pre-motor cortex. Dots are individual observations, and the shaded lines represent the confidence interval of the slopes.

Next, we investigated the interaction of interbrain synchrony X exposure to stressful events, by plotting the interaction at the mean of the moderator (+/- 1 standard deviation). A simple-slope analysis revealed that the effect of exposure to stressful events was the highest when interbrain synchrony was low (estimate = 0.86, p < 0.001), and lower when interbrain synchrony was at the mean (estimate = 0.75, p < 0.001) and when interbrain synchrony was high (estimate = 0.64, p < 0.001). See Fig. 5a.

Finally, we examine the three-way interaction exposure to stressful events X interbrain synchrony X ROI, by looking at the model in each ROI separately. This analysis reveals that while the main effect of exposure to stressful events was significant in each ROI (for full details of the model see Table S5 in the supplementary file), the interaction effect exposure to stressful events X interbrain synchrony was significant only for the left pre-motor area (F = 6.46, p = 0.01). A trend towards significance was shown for the interaction between exposure to stressful events and interbrain synchrony in the right IFG (p = 0.06), the left IFG (p = 0.06), and the right dlPFC (p = 0.07). In the left pre-motor area, the slope of exposure to stressful events is steeper when interbrain synchrony is lower (Fig. 5b).

### The unique contribution of interbrain synchrony over other measures

To test whether interbrain synchrony has a unique contribution to health outcomes, we added PICS as a covariate in models predicting PTSD, general psychiatric, and depressive symptoms. In all cases, the interaction between exposure to stress and interbrain synchrony remained significant, while PICS was independently associated with all symptom variables. However, the three-way interaction with ROI was no longer significant (see Tables S6-S8 in the supplementary file). Additionally, interbrain synchrony and its interaction with ROI were not associated with PICS, suggesting non-overlapping contributions (Supplementary Table S9).

We also tested whether gender moderated the key associations reported in our main models. Women reported more depressive (t = −2.35, p = 0.02) and general psychiatric symptoms (t = −2.51, p = 0.02), but not PTSD (t = −1.54, p = 0.13). Interbrain synchrony levels did not differ by gender (F = 0.35, p = 0.55). Testing gender as a fourth moderator revealed that main effects and lower-order interactions were unchanged, but a significant four-way interaction (Exposure × Interbrain Synchrony × ROI × Gender) indicated that the three-way interaction varied between men and women. Thus, while gender does not affect interbrain synchrony directly, it may shape how interbrain synchrony relates to symptoms in specific ROIs under stress. Full results are in the Supplementary Materials file (Tables S10-S12).

## Discussion

In the present study, we investigated the moderating effect of interbrain synchrony on the relationship between exposure to traumatic events and trauma-related psychopathology. Our findings that exposure to traumatic events is related to increased levels of PTSD, depression, and general psychiatric symptoms replicate the well-established association regarding traumatic events in general [1, 2] and specifically regarding the terror attack in Israel on October 7, 2023 [8].

Critically, we found that spontaneous interbrain synchronization during free conversations prior to exposure to the terror attack moderated the effects of traumatic event exposure. Specifically, individuals showing higher levels of synchronization prior to the traumatic events were less vulnerable to the effects of traumatic exposure. Greater synchronization was associated with a weaker relationship between traumatic exposure and symptoms of PTSD, depression, and general psychiatric conditions.

While interbrain synchrony reflects an emergent property of social interaction contexts/settings, recent evidence suggests that the tendency to synchronize is also an individual characteristic [24, 41–43]. Our study aligns with these findings, emphasizing that individuals differ in their propensity for interpersonal synchrony, which is an important aspect of social adaptation. Notably, our study is the first to demonstrate that the tendency for interbrain synchronization is associated with resilience to stressful events.

Why does the propensity for interbrain synchrony protect against the detrimental effects of traumatic exposure? First, it has been suggested that interpersonal synchrony can foster physiological and emotional regulation [61, 62]. Thus, it is possible that the individuals who tend to synchronize their neural activity are more likely to benefit from the potential of the social environment to regulate their distress [41]. They might also be more likely to help others emotionally regulate themselves, which may provide them with a more emotionally stable environment. In times of high levels of stress and terror, this tendency may provide a protective shield against the adversities of exposure to trauma. Second, interbrain synchrony has been associated with increased closeness and cooperation [39]. While subjective feelings of closeness, such as those captured by PICS scales, are well known to protect against trauma-related psychopathology, our findings suggest that interbrain synchrony provides an additional, objective marker of social engagement that uniquely moderates the effects of traumatic exposure. Importantly, we found no significant relationship between synchrony and PICS, and the main interaction between synchrony and exposure remained significant even when PICS was statistically controlled for. This supports the notion that interbrain synchrony does not merely reflect perceived closeness, but may capture implicit social processes, such as mutual understanding or joint attention, that extend beyond conscious self-report. During periods of adversity, the ability to establish social closeness and engage in cooperative interactions, even with unfamiliar individuals, may serve as a critical adaptive mechanism. Social closeness has the potential to foster a sense of safety and connectedness, which can contribute to emotional regulation and mitigate distress.

In the present study, we examined interbrain synchrony specifically in three regions: the IFG, the premotor cortices, and the dlPFC. While interbrain synchrony in the IFG showed marginally significant effects, we find that interbrain synchrony moderated the effect of exposure to traumatic events most prominently in the pre-motor area. The premotor cortices are considered to be a part of the mirror neuron system, in which observation and execution of action are manifested similarly [63, 64]. Studies that examine interbrain synchrony in this area found that it is associated with prosocial effects. For example, Balconi et al. [65], found an increased interbrain synchrony in the premotor area after a gift exchange, and Liu et al. [66], found an increased interbrain synchrony in the premotor area during a cooperative button-press task. As interbrain synchrony may be associated with similarity in mental processes [40], it seems plausible that individuals who tend to synchronize their activity in these areas, exhibit more emotional empathy in their interactions with others. Thus, by feeling what others feel, they may benefit from feelings of shared fate and togetherness. Indeed, empathy is considered a protective factor from PTSD [67].

Interestingly, it was already found that patients with psychopathology in general and specifically PTSD have difficulties in empathy [68–70]. Patients with PTSD have different neural activity in the observation-execution system compared to healthy individuals [64]. Thus, individuals with a tendency for lower interbrain synchrony may have less empathy, which makes them more susceptible to the adversities of exposure to traumatic events.

Our study has several limitations. First, interbrain synchrony was measured dyadically, while trauma-related psychopathology was assessed individually. While synchrony captures not only individual but also social aspects [43], it is nonetheless predicted vulnerability or resilience to trauma. Notably, while some studies show that interpersonal synchrony has some reliability as an individual trait [41, 42], the reliability of interbrain synchrony specifically as a trait-like remains unestablished. Future studies should assess synchrony across multiple interactions, partners, and time points to better capture individual profiles. Additionally, synchrony was measured in a single experimental interaction with strangers, limiting ecological validity; measuring synchrony in close relationships could improve generalizability. Moreover, while our main hypotheses were theory-driven, the ROI-level analyses were exploratory. As such, findings related to specific brain regions should be interpreted with caution and require replication in future studies. Additionally, the three-way interaction with ROI was not significant when including PICS in the model, indicating less robust findings.

Our sample (19–38 years, 73.5% women, Israeli civilians) may limit generalizability. Prior literature suggests sex differences in brain connectivity and social cognition [71, 72] as well as differences in trauma vulnerability and recovery trajectories [73]. In our data, women reported more depression and general psychopathology, though gender did not directly affect synchrony; however, it moderated the three-way interaction with exposure to stress and ROI. Age and cultural context may also shape these effects [74], highlighting the need for more diverse samples.

Furthermore, while our models were able to explain 22–27% of the variance in outcomes, this indicates that a large portion of the variance remains unexplained. This likely reflects unmeasured confounding factors such as prior trauma history, baseline psychopathology, personality traits, social support, or socioeconomic status. Additionally, the study design is correlational and post-hoc: interbrain synchrony was measured before the trauma ever occurred; however, alternative causal explanations cannot be ruled out. The modest convenience sample (n = 98) lacked a priori power analysis, limiting effect-size precision and increasing Type II error risk, though hypotheses were focused and interpretations cautious. Random intercepts for dyads were included, but random slopes could not be modeled due to convergence issues, potentially inflating Type I error. Finally, reliance on self-report measures may introduce reporting bias [75].

These limitations notwithstanding, our study is the first to provide evidence for an interpersonal biomarker that may protect from the adverse effects of exposure to traumatic events. Our study shows that the tendency for interpersonal brain synchrony moderates the effects of exposure to traumatic events, thus opening up a way for new and promising treatment and prevention programs. It has already been suggested that interbrain synchrony can be enhanced through neurofeedback [76], and our study suggests that such an intervention could potentially help reduce the adversity following traumatic events.

## Supplementary information

Supplementary Material

## Data Availability

The datasets generated and/or analyzed during the current study are available from the corresponding author (O.M.) upon reasonable request.
