# Supplementary material for: Interbrain synchrony and its potential role in modulating the impact of traumatic events
Source: Transl Psychiatry. 2025 Nov 28;16:30. doi: 10.1038/s41398-025-03770-0 (PMC12811243; doi:10.1038/s41398-025-03770-0)
Supplement: Supplementary file 1 — Supplementary Material [file 41398_2025_3770_MOESM1_ESM.docx]

**Supplementary File**

**Exposure to Stressful Events Questionnaire**

Dear Participant,

As you complete the following questionnaire, please reflect on events that took place during the period beginning October 7, 2023. For each situation listed, specify the extent of your exposure.

| **I had exposure to this almost daily/ more than once a day** | **I had exposure to this at least once a week** | **I had exposure to this at least once a month** | **I had a few exposures to this** | **I had a single exposure to this** | **I had no exposure to this** |  |  |
| --- | --- | --- | --- | --- | --- | --- | --- |
| 6 | 5 | 4 | 3 | 2 | 1 | **I was in a place where my life was under serious threat** | **1.** |
| 6 | 5 | 4 | 3 | 2 | 1 | **I was in a situation where my physical well-being was harmed or threatened** | **2.** |
| 6 | 5 | 4 | 3 | 2 | 1 | **I was in a situation where my mental well-being was harmed or threatened** | **3.** |
| 6 | 5 | 4 | 3 | 2 | 1 | **I was required to shelter in my home/safe room/bomb shelter due to a direct and immediate threat** | **4.** |
| 6 | 5 | 4 | 3 | 2 | 1 | **I was in a place that was subjected to direct fire or an attack** | **5.** |
| 6 | 5 | 4 | 3 | 2 | 1 | **I found myself in an area without available shelter during a siren (for example, while driving on a highway)** | **6.** |
| 6 | 5 | 4 | 3 | 2 | 1 | **My personal belongings /property were damaged as a result of the war** | **7.** |
| 6 | 5 | 4 | 3 | 2 | 1 | **I encountered events that disrupted my basic daily functioning** | **8.** |
| 6 | 5 | 4 | 3 | 2 | 1 | **I encountered events that disrupted my ability to pursue important goals in my life** | **9.** |
| 6 | 5 | 4 | 3 | 2 | 1 | **I experienced a loss of basic services (such as water, electricity, or regular food supply)** | **10.** |

**Equation 1**

**Post-Traumatic Stress Symptoms ~ 1 + Exposure to Stressful Events + Interbrain Synchrony + ROI + Days Since Baseline+ Interbrain Synchrony X ROI + Exposure to Stressful Events X ROI + Interbrain Synchrony X Exposure to stressful events + Interbrain Synchrony X Exposure to stressful events X ROI +( 1 | dyad)**

**Equation 2**

**Psychiatric Symptoms ~ 1 + Exposure to Stressful Events + Interbrain Synchrony + ROI + Days Since Baseline+ Interbrain Synchrony X ROI + Exposure to Stressful Events X ROI + Interbrain Synchrony X Exposure to stressful events + Interbrain Synchrony X Exposure to stressful events X ROI +( 1 | dyad)**

**Equation 3**

**Depression Symptoms ~ 1 + Exposure to Stressful Events + Interbrain Synchrony + ROI + Days Since Baseline+ Interbrain Synchrony X ROI + Exposure to Stressful Events X ROI + Interbrain Synchrony X Exposure to stressful events + Interbrain Synchrony X Exposure to stressful events X ROI +( 1 | dyad )**

| **Table S1**  Post-Traumatic Stress Symptoms - Fixed-Effect Estimates | | | | | | | | | |
| --- | --- | --- | --- | --- | --- | --- | --- | --- | --- |
|  | | **F** | | **Num df** | | **Den df** | | **p** | |
| Exposure to Stressful Events |  | 130.39*** |  | 1 |  | 463.9 |  | <.001 ^a^ (< .001) |  |
| Interbrain Synchrony |  | .01 |  | 1 |  | 439.4 |  | .93 |  |
| ROI |  | .06 |  | 5 |  | 408.6 |  | .99 |  |
| Days Since Baseline |  | .28 |  | 1 |  | 57.9 |  | .60 |  |
| Exposure to Stressful Events X ROI |  | .04 |  | 5 |  | 406.5 |  | .99 |  |
| Interbrain Synchrony X ROI |  | .06 |  | 5 |  | 420.6 |  | .99 |  |
| Exposure to Stressful Events X Interbrain Synchrony |  | 5.36* |  | 1 |  | 423.3 |  | .03^a^ (.02) |  |
| Exposure to Stressful Events X Interbrain Synchrony X ROI |  | 1.74 |  | 5 |  | 416.5 |  | .12 ^a^ (.12) |  |

Note: ROI= Regions of interest. a - Corrected using false discovery rate. The original p values are in parentheses. * p <.05., ** ,p < .01. ***, p < .001.

| **Table S2**  Psychiatric Symptoms -Fixed-Effect Estimates | | | | | | | | | | | | | | | | | | |
| --- | --- | --- | --- | --- | --- | --- | --- | --- | --- | --- | --- | --- | --- | --- | --- | --- | --- | --- |
|  | | **F** | | | | **Num df** | | | | **Den df** | | | | **p** | | | | |
| Exposure to Stressful Events |  | | 149.75*** | |  | | 1 | |  | | 463.0 | |  | | | >.001^a^ (< .001) | |  |
| Interbrain Synchrony |  | | .00 |  | | 1 | |  | | 435.6 | |  | | | .95 | |  |  |
| ROI |  | | .03 |  | | 5 | |  | | 406.4 | |  | | | 1.00 | |  |  |
| Days Since Baseline |  | | .00 |  | | 1 | |  | | 56.3 | |  | | | .99 | |  |  |
| Exposure to Stressful Events X ROI |  | | .03 |  | | 5 | |  | | 404.4 | |  | | | .99 | |  |  |
| Interbrain Synchrony X ROI |  | | .09 |  | | 5 | |  | | 417.6 | |  | | | .99 | |  |  |
| Exposure to Stressful Events X Interbrain Synchrony |  | | 4.28* |  | | 1 | |  | | 420.1 | |  | | | .04 ^a^ (.04) | |  |  |
| Exposure to Stressful Events X Interbrain Synchrony X ROI |  | | 2.44* |  | | 5 | |  | | 413.7 | |  | | | .04 ^a (^.03) | |  |  |

Note: ROI= Regions of interest. a - Corrected using false discovery rate. The original p values are in parentheses. * p <.05., ** ,p < .01. ***, p < .001.

**Table S3**

Psychiatric Symptoms - Separate model in each ROI.

|  | df1 | df2 | F.ratio | p.value |
| --- | --- | --- | --- | --- |
| **ROI = dlPFC_L:** | | | | |
| Exposure to Stressful Events | 1 | 432.57 | 41.38 | <.001^a (^<.001) |
| Interbrain Synchrony | 1 | 422.69 | .25 | .62 |
| Days Since Baseline | 1 | 58.17 | .00 | .99 |
| Exposure to Stressful Events X Interbrain Synchrony | 1 | 414.20 | 1.341 | .25 ^a^ (.25) |
| **ROI = dlPFC_R:** | | | | |
| Exposure to Stressful Events | 1 | 431.79 | 49.67 | <.001^a (^<.001) |
| Interbrain Synchrony | 1 | 417.36 | .02 | .89 |
| Days Since Baseline | 1 | 58.17 | .00 | .99 |
| Exposure to Stressful Events X Interbrain Synchrony | 1 | 419.60 | 2.61 | .11 ^a^ (.11) |
| **ROI = IFG_L:** | | | | |
| Exposure to Stressful Events | 1 | 433.75 | 46.44 | <.001^a (^<.001) |
| Interbrain Synchrony | 1 | 429.55 | .09 | .77 |
| Days Since Baseline | 1 | 58.17 | .00 | .99 |
| Exposure to Stressful Events X Interbrain Synchrony | 1 | 423.81 | 3.39 | .07 ^a^ (.07) |
| **ROI = IFG_R:** | | | | |
| Exposure to Stressful Events | 1 | 431.72 | 44.20 | <.001^a (^<.001) |
| Interbrain Synchrony | 1 | 428.07 | .06 | .82 |
| Days Since Baseline | 1 | 58.17 | .00 | .99 |
| Exposure to Stressful Events X Interbrain Synchrony | 1 | 415.11 | 2.96 | .09 ^a^ (.09) |
| **ROI = preMotor_L:** | | | | |
| Exposure to Stressful Events | 1 | 429.92 | 51.94 | <.001^a (^<.001) |
| Interbrain Synchrony | 1 | 417.63 | .00 | .98 |
| Days Since Baseline | 1 | 58.17 | .00 | .99 |
| Exposure to Stressful Events X Interbrain Synchrony | 1 | 415.96* | 5.07 | .02 ^a^ (.02) |
| **ROI = preMotor_R:** | | | | |
| Exposure to Stressful Events | 1 | 431.76 | 49.61 | <.001^a (^<.001) |
| Interbrain Synchrony | 1 | 416.51 | 04 | .85 |
| Days Since Baseline | 1 | 58.17 | .00 | .99 |
| Exposure to Stressful Events X Interbrain Synchrony | 1 | 412.40 | 2.02 | .16 ^a^ (.16) |

Note: ROI = Region of interest, dlPFC_L = The left dorsolateral pre-frontal cortex, dlPFC_R = The right dorsolateral pre-frontal cortex, IFG_L = The left inferior frontal gyrus, IFG_R = The right inferior frontal gyrus, preMotor_L = The left pre-motor cortex, preMotor_R = The right pre-motor cortex. a - Corrected using false discovery rate. The original p values are in parentheses. * p <.05., ** ,p < .01. ***, p < .001.

| **Table S4**  Depression Symptoms - Fixed-Effect Estimates | | | | | | | | | |
| --- | --- | --- | --- | --- | --- | --- | --- | --- | --- |
|  | | **F** | | **Num df** | | **Den df** | | **p** | |
| Exposure to Stressful Events |  | 177.94*** |  | 1 |  | 461.9 |  | >.001^a^ (< .001) |  |
| Interbrain Synchrony |  | .03 |  | 1 |  | 434.8 |  | .86 |  |
| ROI |  | .04 |  | 5 |  | 408.0 |  | .99 |  |
| Days Since Baseline |  | .03 |  | 1 |  | 58.2 |  | .88 |  |
| Exposure to Stressful Events X ROI |  | .05 |  | 5 |  | 406.2 |  | .99 |  |
| Interbrain Synchrony X ROI |  | .06 |  | 5 |  | 418.2 |  | .99 |  |
| Exposure to Stressful Events X Interbrain Synchrony |  | 6.35* |  | 1 |  | 420.5 |  | .02 ^a^  (.01) |  |
| Exposure to Stressful Events X Interbrain Synchrony X ROI |  | 2.45* |  | 5 |  | 414.6 |  | .03 ^a^ (.03) |  |

Note: ROI= Regions of interest. a - Corrected using false discovery rate. The original p values are in parentheses. * p <.05., ** ,p < .01. ***, p < .001.

**Table S5**

Depression Symptoms - Separate model in each ROI.

|  | df1 | df2 | F.ratio | p.value |
| --- | --- | --- | --- | --- |
| **ROI = dlPFC_L:** | | | | |
| Exposure to Stressful Events | 1 | 431.13 | 49.92 | <.001^a (^<.001) |
| Interbrain Synchrony | 1 | 421.62 | .23 | .63 |
| Days Since Baseline | 1 | 58.29 | .02 | .88 |
| Exposure to Stressful Events X Interbrain Synchrony | 1 | 413.62 | .89 | .35 ^a^ (.35) |
| **ROI = dlPFC_R:** | | | | |
| Exposure to Stressful Events | 1 | 430.38 | 58.52 | <.001^a (^<.001) |
| Interbrain Synchrony | 1 | 416.51 | .04 | .84 |
| Days Since Baseline | 1 | 58.29 | .02 | .88 |
| Exposure to Stressful Events X Interbrain Synchrony | 1 | 418.73 | 3.30 | .07 ^a^ (.07) |
| **ROI = IFG_L:** | | | | |
| Exposure to Stressful Events | 1 | 432.28 | 56.18 | <.001^a (^<.001) |
| Interbrain Synchrony | 1 | 428.26 | .02 | .88 |
| Days Since Baseline | 1 | 58.29 | .02 | .88 |
| Exposure to Stressful Events X Interbrain Synchrony | 1 | 422.70 | 3.66 | .06 ^a^ (.06) |
| **ROI = IFG_R:** | | | | |
| Exposure to Stressful Events | 1 | 430.34 | 51.31 | <.001^a (^<.001) |
| Interbrain Synchrony | 1 | 426.77 | .03 | .87 |
| Days Since Baseline | 1 | 58.29 | .02 | .88 |
| Exposure to Stressful Events X Interbrain Synchrony | 1 | 414.45 | 3.577 | .06 ^a^ (.06) |
| **ROI = preMotor_L:** | | | | |
| Exposure to Stressful Events | 1 | 428.59 | 63.45 | <.001^a (^<.001) |
| Interbrain Synchrony | 1 | 416.78 | .00 | .99 |
| Days Since Baseline | 1 | 58.29 | .02 | .88 |
| Exposure to Stressful Events X Interbrain Synchrony | 1 | 415.25* | 6.46 | .01 ^a^ (.01) |
| **ROI = preMotor_R:** | | | | |
| Exposure to Stressful Events | 1 | 430.35 | 59.08 | <.001^a (^<.001) |
| Interbrain Synchrony | 1 | 415.72 | .03 | .86 |
| Days Since Baseline | 1 | 58.29 | .02 | .88 |
| Exposure to Stressful Events X Interbrain Synchrony | 1 | 411.90 | 1.40 | .24 ^a^ (.24) |

Note: ROI = Region of interest, dlPFC_L = The left dorsolateral pre-frontal cortex, dlPFC_R = The right dorsolateral pre-frontal cortex, IFG_L = The left inferior frontal gyrus, IFG_R = The right inferior frontal gyrus, preMotor_L = The left pre-motor cortex, preMotor_R = The right pre-motor cortex. a - Corrected using false discovery rate. The original p values are in parentheses. * p <.05., ** ,p < .01. ***, p < .001.

**Additional Analyses**

**Table S6**

| Post-Traumatic Stress Symptoms - Fixed-Effect Estimates | | | | | | | | | |
| --- | --- | --- | --- | --- | --- | --- | --- | --- | --- |
|  | | **F** | | **Num df** | | **Den df** | | **p** | |
| Exposure to Stressful Events |  | 109.52*** |  | 1 |  | 440.0 |  | < .001 |  |
| Interbrain Synchrony |  | .01 |  | 1 |  | 414.2 |  | .90 |  |
| ROI |  | .04 |  | 5 |  | 384.8 |  | .99 |  |
| PICS |  | 32.13*** |  | 1 |  | 427.5 |  | < .001 |  |
| Days Since Baseline |  | .07 |  | 1 |  | 53.9 |  | .79 |  |
| Interbrain Synchrony X ROI |  | .05 |  | 5 |  | 396.0 |  | .99 |  |
| Exposure to Stressful Events X Interbrain Synchrony |  | 5.90* |  | 1 |  | 400.1 |  | .02 |  |
| Exposure to Stressful Events X ROI |  | .03 |  | 5 |  | 382.6 |  | 1.00 |  |
| Exposure to Stressful Events X Interbrain Synchrony X ROI |  | 1.30 |  | 5 |  | 392.1 |  | .26 |  |
| Note: ROI = Regions of interest. * p <.05., ** ,p < .01. ***, p < .001. | | | | | | | | | |
|  | | | | | | | | | |

| **Table S7**  Psychiatric Symptoms -Fixed-Effect Estimates | | | | | | | | | |
| --- | --- | --- | --- | --- | --- | --- | --- | --- | --- |
|  | | **F** | | **Num df** | | **Den df** | | **p** | |
| Exposure to Stressful Events |  | 133.41*** |  | 1 |  | 438.5 |  | < .001 |  |
| Interbrain Synchrony |  | .01 |  | 1 |  | 408.9 |  | .93 |  |
| ROI |  | .02 |  | 5 |  | 383.0 |  | 1.000 |  |
| Days Since Baseline |  | .08 |  | 1 |  | 53.1 |  | .78 |  |
| PICS |  | 59.73*** |  | 1 |  | 436.3 |  | < .001 |  |
| Interbrain Synchrony X ROI |  | .06 |  | 5 |  | 392.8 |  | .99 |  |
| Exposure to Stressful Events X Interbrain Synchrony |  | 5.12* |  | 1 |  | 396.4 |  | .02 |  |
| Exposure to Stressful Events X ROI |  | .05 |  | 5 |  | 381.2 |  | .999 |  |
| Exposure to Stressful Events X Interbrain Synchrony X ROI |  | 1.56 |  | 5 |  | 389.3 |  | .17 |  |

Note: ROI = Regions of interest. * p <.05., ** ,p < .01. ***, p < .001.

| **Table S8**  Depression Symptoms - Fixed-Effect Estimates | | | | | | | | | |
| --- | --- | --- | --- | --- | --- | --- | --- | --- | --- |
|  | | **F** | | **Num df** | | **Den df** | | **p** | |
| Exposure to Stressful Events |  | 168.18*** |  | 1 |  | 438.9 |  | < .001 |  |
| Interbrain Synchrony |  | .05 |  | 1 |  | 410.7 |  | .83 |  |
| ROI |  | .03 |  | 5 |  | 385.0 |  | 1.00 |  |
| Days Since Baseline |  | .11 |  | 1 |  | 54.9 |  | .75 |  |
| PICS |  | 45.05*** |  | 1 |  | 435.6 |  | < .001 |  |
| Interbrain Synchrony X ROI |  | .05 |  | 5 |  | 394.7 |  | .99 |  |
| Exposure to Stressful Events X ROI |  | .04 |  | 5 |  | 383.1 |  | .99 |  |
| Exposure to Stressful Events X Interbrain Synchrony |  | 6.50* |  | 1 |  | 398.3 |  | .01 |  |
| Exposure to Stressful Events X Interbrain Synchrony X ROI |  | 1.49 |  | 5 |  | 391.3 |  | .19 |  |
| Note: ROI = Regions of interest. * p <.05., ** ,p < .01. ***, p < .001. | | | | | | | | | |

| **Table S9**  Perceived Interpersonal Closeness - Fixed-Effect Estimates | | | | | | | | | |
| --- | --- | --- | --- | --- | --- | --- | --- | --- | --- |
|  | | **F** | | **Num df** | | **Den df** | | **p** | |
| Interbrain Synchrony |  | .01 |  | 1 |  | 420.1 |  | .94 |  |
| ROI |  | .00 |  | 5 |  | 398.5 |  | 1.00 |  |
| Days |  | .20 |  | 1 |  | 56.8 |  | .65 |  |
| Interbrain Synchrony X ROI |  | .01 |  | 5 |  | 406.1 |  | 1.00 |  |
| Note: ROI = Regions of interest. | | | | | | | | | |
|  | | | | | | | | | |

| **Table S10**  Post-Traumatic Stress Symptoms - Fixed-Effect Estimates | | | | | | | | | |
| --- | --- | --- | --- | --- | --- | --- | --- | --- | --- |
|  | | **F** | | **Num df** | | **Den df** | | **p** | |
| Exposure to Stressful Events |  | 106.28*** |  | 1 |  | 426.3 |  | < .001 |  |
| Interbrain Synchrony |  | .00 |  | 1 |  | 408.2 |  | .96 |  |
| ROI |  | .13 |  | 5 |  | 380.1 |  | .99 |  |
| Days |  | .18 |  | 1 |  | 57.4 |  | .68 |  |
| Gender |  | 2.46 |  | 1 |  | 57.9 |  | .12 |  |
| Interbrain Synchrony X ROI |  | .03 |  | 5 |  | 397.6 |  | .99 |  |
| Exposure to Stressful Events X Interbrain Synchrony |  | 9.73** |  | 1 |  | 395.0 |  | .00 |  |
| Exposure to Stressful Events X ROI |  | .17 |  | 5 |  | 374.6 |  | .97 |  |
| ROI X Gender |  | .04 |  | 5 |  | 380.2 |  | .99 |  |
| Exposure to Stressful Events X Gender |  | 2.78 |  | 1 |  | 427.0 |  | .10 |  |
| Interbrain Synchrony X Gender |  | .37 |  | 1 |  | 405.8 |  | .54 |  |
| Exposure to Stressful Events X Interbrain Synchrony X ROI |  | 2.16 |  | 5 |  | 386.9 |  | .06 |  |
| Exposure to Stressful Events X ROI X Gender |  | .40 |  | 5 |  | 374.6 |  | .85 |  |
| Interbrain Synchrony X ROI X Gender |  | .12 |  | 5 |  | 397.5 |  | .99 |  |
| Exposure to Stressful Events X Interbrain Synchrony X Gender |  | 1.60 |  | 1 |  | 395.1 |  | .21 |  |
| Interbrain Synchrony X ROI X Days X Gender |  | .09 |  | 12 |  | 390.1 |  | 1.00 |  |
| Exposure to Stressful Events X Interbrain Synchrony X ROI X Gender |  | 2.27* |  | 5 |  | 386.9 |  | .05 |  |
| Note: ROI = Regions of interest. * p <.05., ** ,p < .01. ***, p < .001. | | | | | | | | | |
|  | | | | | | | | | |

| **Table S11**  Psychiatric Symptoms -Fixed-Effect Estimates | | | | | | | | | |
| --- | --- | --- | --- | --- | --- | --- | --- | --- | --- |
|  | | **F** | | **Num df** | | **Den df** | | **p** | |
| Exposure to Stressful Events |  | 128.93*** |  | 1 |  | 439.1 |  | < .001 |  |
| Interbrain Synchrony |  | .00 |  | 1 |  | 417.2 |  | .99 |  |
| ROI |  | .13 |  | 5 |  | 387.3 |  | .99 |  |
| Days |  | .00 |  | 1 |  | 55.6 |  | 1.00 |  |
| Gender |  | 9.68** |  | 1 |  | 56.4 |  | .00 |  |
| Interbrain Synchrony X ROI |  | .07 |  | 5 |  | 404.5 |  | .99 |  |
| Exposure to Stressful Events X Interbrain Synchrony |  | 9.08** |  | 1 |  | 408.8 |  | .00 |  |
| Exposure to Stressful Events X ROI |  | .23 |  | 5 |  | 384.0 |  | .95 |  |
| ROI X Gender |  | .08 |  | 5 |  | 387.3 |  | .99 |  |
| Interbrain Synchrony X Gender |  | .24 |  | 1 |  | 416.3 |  | .63 |  |
| Exposure to Stressful Events X Gender |  | 3.08 |  | 1 |  | 439.6 |  | .08 |  |
| Exposure to Stressful Events X Interbrain Synchrony X ROI |  | 2.88* |  | 5 |  | 398.2 |  | .01 |  |
| Interbrain Synchrony X ROI X Gender |  | .11 |  | 5 |  | 404.4 |  | .99 |  |
| Exposure to Stressful Events X Interbrain Synchrony X Gender |  | .43 |  | 1 |  | 408.6 |  | .51 |  |
| Exposure to Stressful Events X ROI X Gender |  | .61 |  | 5 |  | 384.0 |  | .69 |  |
| Exposure to Stressful Events X Interbrain Synchrony X ROI X Gender |  | 2.39* |  | 5 |  | 398.2 |  | .04 |  |
| Note: ROI = Regions of interest. * p <.05., ** ,p < .01. ***, p < .001. | | | | | | | | | |
|  | | | | | | | | | |

| **Table S12**  Depression Symptoms - Fixed-Effect Estimates | | | | | | | | | |
| --- | --- | --- | --- | --- | --- | --- | --- | --- | --- |
|  | | **F** | | **Num df** | | **Den df** | | **p** | |
| Exposure to Stressful Events |  | 154.99*** |  | 1 |  | 439.7 |  | < .001 |  |
| Interbrain Synchrony |  | .00 |  | 1 |  | 416.8 |  | .96 |  |
| ROI |  | .15 |  | 5 |  | 388.6 |  | .98 |  |
| Days |  | .00 |  | 1 |  | 57.4 |  | .95 |  |
| Gender |  | 10.02** |  | 1 |  | 58.1 |  | .00 |  |
| Interbrain Synchrony X ROI |  | .07 |  | 5 |  | 404.6 |  | .99 |  |
| Exposure to Stressful Events X ROI |  | .41 |  | 5 |  | 385.5 |  | .84 |  |
| Exposure to Stressful Events X Interbrain Synchrony |  | 13.92*** |  | 1 |  | 408.9 |  | < .001 |  |
| ROI X Gender |  | .07 |  | 5 |  | 388.6 |  | .99 |  |
| Interbrain Synchrony X Gender |  | .10 |  | 1 |  | 415.8 |  | .75 |  |
| Exposure to Stressful Events X Gender |  | 14.63*** |  | 1 |  | 439.9 |  | < .001 |  |
| Exposure to Stressful Events X Interbrain Synchrony X ROI |  | 3.36** |  | 5 |  | 398.8 |  | .01 |  |
| Interbrain Synchrony X ROI X Gender |  | .09 |  | 5 |  | 404.6 |  | .99 |  |
| Exposure to Stressful Events X ROI X Gender |  | .63 |  | 5 |  | 385.5 |  | .68 |  |
| Exposure to Stressful Events X Interbrain Synchrony X Gender |  | 1.36 |  | 1 |  | 408.7 |  | .24 |  |
| Exposure to Stressful Events X Interbrain Synchrony X ROI X Gender |  | 3.61** |  | 5 |  | 398.8 |  | .00 |  |
| Note: ROI = Regions of interest. * p <.05., ** ,p < .01. ***, p < .001. | | | | | | | | | |
|  | | | | | | | | | |
